# A High-Affinity CDR-Grafted Antibody against Influenza A H5N1 Viruses Recognizes a Conserved Epitope of H5 Hemagglutinin

**DOI:** 10.1371/journal.pone.0088777

**Published:** 2014-02-18

**Authors:** Feifei Xiong, Liliang Xia, Jingfang Wang, Biao Wu, Dengyu Wang, Longfang Yuan, Yating Cheng, Hongying Zhu, Xiaoyan Che, Qinghua Zhang, Guoping Zhao, Ying Wang

**Affiliations:** 1 School of Life Science and Technology, Tongji University, Shanghai, China; 2 Shanghai-MOST Key Laboratory of Health and Disease Genomics, Chinese National Human Genome Center at Shanghai, Shanghai, China; 3 Shanghai Center for Systems Biomedicine, Shanghai Jiaotong University, Shanghai, China; 4 Central Laboratory, Zhujiang Hospital, The Southern Medical University, Guangzhou, China; 5 Shanghai Institute of Immunology, Shanghai Jiaotong University School of Medicine, Shanghai, China; National Cancer Institute, NIH, United States of America

## Abstract

Highly pathogenic avian influenza (HPAI) H5N1 virus infection is still a potential threat to public health worldwide. While vaccines and antiviral drugs are currently under development, neutralizing antibodies could offer an alternative strategy to prevent and treat H5N1 virus infection. In the present study, we had developed a humanized antibody against H5N1 viruses from mouse-derived hybridoma in order to minimize its immunogenicity for potential clinical application. The humanized antibody hH5M9 was generated by transferring the mouse complementarity determining region (CDR) residues together with four key framework region (FR) residues onto the FR of the human antibody. This humanized antibody exhibited high affinity and specificity comparable to the parental mouse or chimeric counterpart with broad and strong neutralization activity against all H5N1 clades and subclades except for Egypt clades investigated. Furthermore, through epitope mapping we identified a linear epitope on the top region of hemagglutinin (HA) that was H5N1 specific and conserved. Our results for the first time reported a humanized antibody against H5N1 viruses by CDR grafting method. With the expected lower immunogenicity, this humanized antibody was expected to be more efficacious than murine or human-mouse chimeric antibodies for future application in humans.

## Introduction

H5N1 virus, one of highly pathogenic avian influenza (HPAI) strains, has caused numerous outbreaks in poultry in Southeast Asia since 1997 [1]–[3], and more recently continues to spread globally. These outbreaks are accompanied by the occasional transmission of HPAI H5N1 virus to humans, resulting in a total of 628 cases with 374 deaths in 15 countries since 2003 [4].

To prevent H5N1 pandemic, world-wide efforts have been made to develop and stockpile preventive vaccines, antiviral drugs as well as passive immune therapies [5], [6]. Vaccine strategies have been found to be only effective at preventive stage whereas they can be easily hindered by antigenic variation of the influenza strains [5]. Antiviral treatment is an ideal method. But currently available options are limited [6]. Antibody-based treatment has been successfully used prophylactically against many virus-infected diseases, such as those caused by hepatitis A virus, hepatitis B virus, cytomegalovirus, rabies virus, varicella virus and respiratory syncytial virus infection [7]. Thus, it is feasible to induce humoral immunity in humans through preventive vaccination and neutralizing antibody generation to protect against H5N1 virus infection. In addition, passive immune therapies have been further highlighted by transfusion of human convalescent sera leading to a 50% reduction in influenza mortality during the 1918 Spanish influenza pandemic, and more recently by anecdotal reports of treating H5N1 human infection with convalescent sera in China [8], [9].

Influenza hemagglutinin (HA), with 16 antigenic distinct subtypes, is the main target for neutralizing antibodies against influenza viral infections [10]–[16]. Three HA monomers, each consisting of one HA1 and one HA2 subunit, form the HA molecule on the surface of influenza virion. The HA1 subunit, globular head domain of HA molecule, is the most immunogenic region of the HA protein, containing the receptor binding site which mediates viral attachment to the host cell membrane. The HA2 subunit constitutes the core fusion machinery in the stalk region [17], [18]. Several research groups have reported that monoclonal antibodies (mAbs) against the HA protein of the influenza virus could confer prophylactic and therapeutic protection in mouse models [14], [15], [19]–[21].

Although antibody-based therapy has displayed the potential prevention and treatment of H5N1 virus infection in human, the efficacy of murine mAbs is hampered by their diminished serum half-life, inability to trigger human effector functions and the induction of a human anti-mouse antibody (HAMA) response [22], [23]. To counter these problems, several strategies have been devised including the generation of chimeric, humanized or human antibodies. CDR grafting is a way to humanize murine antibodies by grafting the complementarity determining regions (CDRs) of a murine mAb onto the framework regions (FRs) of a human antibody while retaining those rodent FR residues that influence antigen-binding activity [24]–[26]. Compared with chimeric antibodies, CDR-grafted antibodies possess lower immunogenicity with more successful application in clinic [27]–[29].

In the previous study, the mouse monoclonal antibody H5M9 (mH5M9) demonstrated broad and strong neutralizing activity against H5N1 viruses isolated from 1997 to 2006 [30]. Accordingly, here we described the generation and characterization of a CDR-grafted anti-H5N1 antibody derived from mH5M9. We further identified a linear epitope on the top of HA globular region recognized by the engineering antibody, which was highly conserved in different clades of currently epidemic avian H5N1 viruses. The development of this CDR-grafted antibody might provide an alternative strategy in prevention and treatment of human H5N1 virus infection.

## Results

### Generation of chimeric and CDR-grafted antibodies against HA of H5N1 viruses

**Figure 1:**
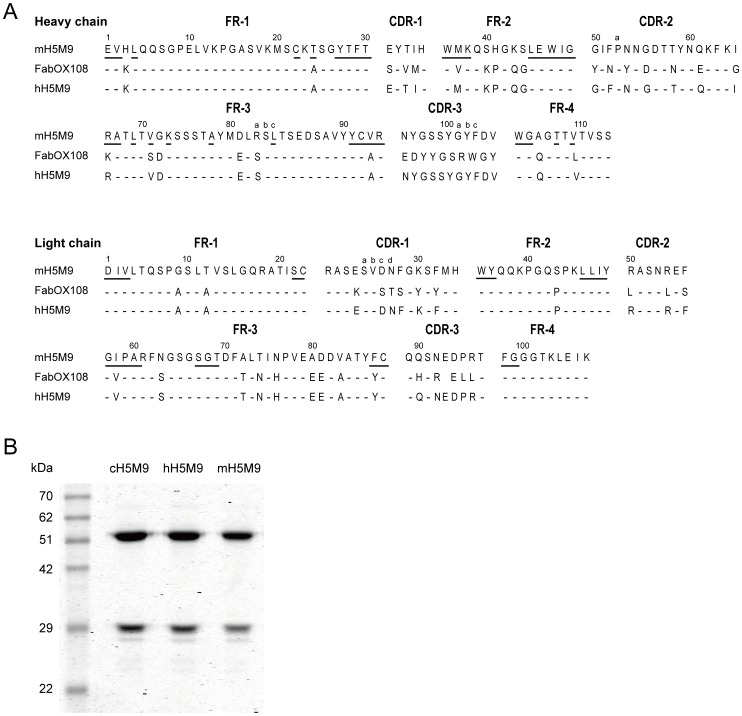
Generation of chimeric cH5M9 and CDR-grafted hH5M9 antibodies. (A) Amino acid sequences of the VH regions of mouse antibody mH5M9, human antibody FabOX108 and CDR-grafted antibody hH5M9. Amino acid residues were listed according to the convention of Kabat et al. [31]. Residues shown by underline in frameworks were deemed essential for maintaining the combining sites of mH5M9. Dashes indicated residues that were identical in mH5M9, FabOX108 and hH5M9 whereas gaps denoted amino acid residues missing at those positions. (B) SDS-PAGE analysis of purified cH5M9 and hH5M9 under reducing conditions. mH5M9 was used as the positive control.

To generate a human-mouse chimeric antibody, the variable regions of both light chain (VL) and heavy chain (VH) from mH5M9 were subcloned into IgG expression cassette vectors IFH and IFL, respectively. IFH-VH and IFL-VL vectors were co-expressed in 293F cells, yielding chimeric H5M9 (cH5M9) (Figure 1B).

For the construction of CDR-grafted H5M9 antibody, a BLASTP-search against the non-redundant protein database was performed to screen the template for further humanization. The results showed that VH and VL domains of human antibody FabOX108 (PDB No. 3DGG_B and No. 3DGG_A, respectively) were the most homologous to that of mH5M9, respectively (Figure 1A). We thus selected the FRs of this template for grafting CDR residues of mH5M9. Genes from three CDRs of mH5M9 VH/VL regions were directly grafted onto human antibody FabOX108 VH/VL frameworks to generate CDR-grafted antibody gene. Meanwhile, four FR residues of mH5M9 VH region (Met^37^, Arg^66^, Va^l71^, Val^109^) were retained, which were deduced to influence the conformations of CDRs according to the simulation of three dimensional structure of VH and VL regions (Figure S2). The recombinant CDR-grafted VH and VL genes were cloned into IgG expression cassette vectors and co-expressed in 293F cells to obtain a CDR-grafted antibody, named hH5M9 (Figure 1B).

The amino acid residues of the variable regions of mH5M9, CDR-grafted hH5M9 and FabOX108 antibodies were listed in Figure 1A, which were numbered according to the convention of Kabat et al. [31].

### Affinity Determination of chimeric and CDR-grafted H5M9 antibodies

**Figure 2:**
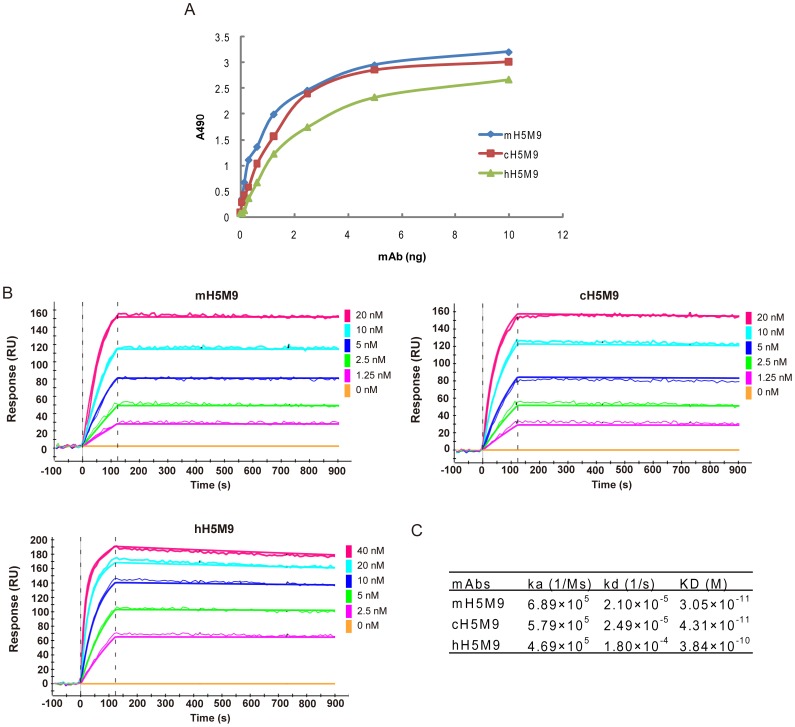
Characterization of cH5M9 and hH5M9 antibodies. (A) Antigen-binding capacity of mH5M9, cH5M9 and hH5M9 were measured by an indirect ELISA. (B) Affinity determination of mH5M9, cH5M9 and hH5M9 by surface plasmon resonance (SPR) assay. Various concentrations of mH5M9, cH5M9, hH5M9 and irrelevant anti-EGFR antibody C225 were injected on a GLC sensor chip immobilizing H5 HA (A/Vietnam/1194/04) for SPR kinetic binding analysis. Binding curves were recorded using ProteOn system in SPR biosensor instrument (BioRad Labs). (C) Calculated values of the association rate constant (*Ka*), dissociation rate constant (*Kd*), and equilibrium dissociation constant (*KD*) of mH5M9, cH5M9 and hH5M9.

An indirect ELISA was first used to analyze the antigen-binding activity of cH5M9 and hH5M9 against H5 HA (A/Vietnam/1194/04). As shown in Figure 2A, cH5M9 had the similar antigen-binding activity to mH5M9, while the antigen-binding activity of hH5M9 was lower than that of mH5M9, suggesting that the humanization of recombinant antibody by CDR grafting might result in the structural alteration of the antigen-binding site more or less and the subsequent decrease of antigen-binding capacity [32].

To further quantify the binding ability of the engineering cH5M9 and hH5M9 antibodies, kinetic binding constants were determined by ProteON surface plasmon resonance (SPR) assay using H5 HA (A/Vietnam/1194/04) coated biosensor. According to the association and dissociation curves of antibodies shown in Figure 2B, the equilibrium dissociation constant (*KD*) values of mH5M9, cH5M9 and hH5M9 were calculated to be 3.05×10^−11^, 4.31×10^−11^ and 3.84×10^−10^ M, respectively (Figure 2C). Although there was no significant difference among the association rate constants (*Ka*) of cH5M9, hH5M9 or mH5M9, the dissociation rate constant (*Kd*) of hH5M9 was slightly faster than other two antibodies, which implied that the substitution of certain residues in antigen-binding sites of VH or VL domains in hH5M9 reduced the binding stability after CDR grafting when compared with its parental or chimeric counterpart.

### Neutralization breadths and potencies of chimeric and CDR-grafted antibodies *in vitro*

In the previous study, mH5M9 showed broad and strong neutralization activity against H5N1 viruses [30]. We also performed hemagglutination inhibition (HI) assays against a panel of HA strains, including H1, H7, H9, and H5, to determine the neutralization activities of cH5M9 and hH5M9 antibodies. cH5M9 and hH5M9 inhibited HAs of all five H5N1 strains tested in a concentration between 7.81 and 15.62 µg/ml whereas failed to inhibit hemagglutination of H1, H7 and H9, exhibiting similar specificity to mH5M9 (Table 1).

**Table 1:** HI titers of three antibodies against Influenza A viruses.

Influenza A Virus	Subtype	Genetic clade	Antibody concentration (µg/ml)		
			mH5M9	cH5M9	hH5M9
A/goose/Guangdong/1/96	H5N1	0	7.81	7.81	15.62
A/Vietnam/1194/04	H5N1	1	7.81	7.81	15.62
A/duck/Anhui/1/06	H5N1	2.3	7.81	7.81	15.62
A/Anhui/1/05	H5N1	2.3.4	7.81	7.81	15.62
A/chicken/Shanxi/2/06	H5N1	7	7.81	7.81	15.62
A/New Caledonia/20/99	H1N1		>250	>250	>250
A/duck/Guangdong/1/96	H7N3		>250	>250	>250
A/chicken/Shandong/6/96	H9N2		>250	>250	>250

To further determine the neutralization breadths and potencies of cH5M9 and hH5M9, we had chosen nine H5 HAs sub-classified into clades 0, 1, 2 and 4 for further titration, which had been associated with human or avian pathogenesis to date (Table 2). cH5M9 and hH5M9 possessed the similar neutralization activity as mH5M9 against all of the H5N1 clades tested, ranging from 0.1 to 12.5 ng/ml, except for clade 2.2.1 virus strain A/Egypt/N05056/09.

**Table 2:** Neutralization activity of three antibodies against H5N1 viruses.

H5N1 virus	Genetic clade	Antibody concentration (ng/ml)		
		mH5M9	cH5M9	hH5M9
A/duck/Hong Kong/p46/97	0	0.2	0.2	0.39
A/Vietnam/1194/04	1	0.2	0.2	0.39
A/Indonesia/5/05	2.1.3	0.049	0.1	0.39
A/Xinjiang/1/06	2.2	0.2	0.39	0.78
A/Egypt/N05056/09	2.2.1	>2000	>2000	>2000
A/Anhui/1/05	2.3.4	0.049	0.049	0.2
A/common magpie/Hong Kong/2256/06	2.3.4	0.1	0.1	0.2
A/Japanese white-eye/Hong Kong/1038/06	2.3.4	0.1	0.1	0.2
A/goose/Guiyang/337/06	4	3.12	3.12	12.5

These result indicated that CDR-grafted hH5M9 might recognize a shared epitope on HAs from almost all clades and subclades of the H5 subtype that was not shared by HAs of H1, H7, and H9 subtypes.

### Epitope mapping

**Figure 3:**
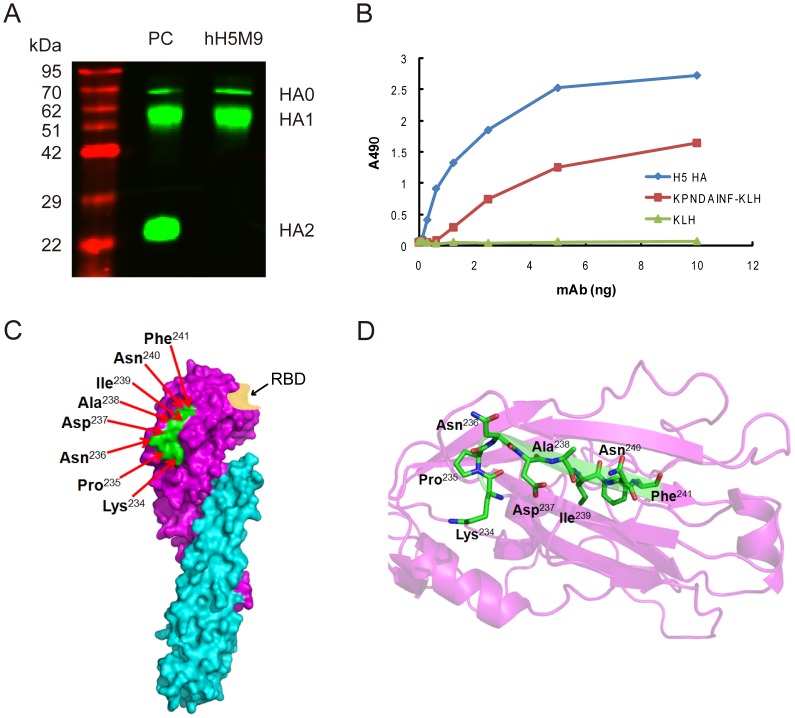
Epitope mapping of hH5M9. (A) Western blotting analysis of hH5M9 with HA. Purified A/Anhui/1/05 HA was applied to SDS-PAGE under reducing conditions. The hH5M9 was used as primary antibody. A rabbit polyclonal antibody against A/Anhui/1/05 was used as positive control (PC). (B) Binding analysis of synthesized epitope “KPNDAINF” with hH5M9 was measured by an indirect ELISA. (C) Schematic representation of the epitope recognized by hH5M9. The linear epitope (KPNDAINF, amino acids 234–241) on the three dimensional structure of the H5 HA (VN1194) mono-structure (PDB No. 2IBX) was colored in green while the receptor binding domain (RBD) was highlighted in yellow. (D) Cartoon illustration of the three dimensional structure of the linear epitope for hH5M9.

To anchor the epitope recognized by hH5M9, two fragments of HA, HA1 and HA2, were first prepared and adapted for epitope mapping. Through western blotting analysis, it was demonstrated that hH5M9 antibody reacted with the viral HA1 protein, rather than HA2. Considering the denaturation of HA1 during the western blotting analysis, the epitope targeted by hH5M9 appeared to be linear rather than conformational (Figure 3A). According to our above results that hH5M9 displayed broad cross-reactivity to H5N1 HAs but not A/Egypt/N05056/09 (Table 2), we first aligned HA1 amino acid sequences of these nine H5N1 isolates used in the binding assay to find out the unique amino acid residues of HA1 of A/Egypt/N05056/09 (Table 3). We found that Arg^22^, Asn^120^, Thr^151^, Gln^152^, Asp^154^, Ile^210^ and Ser^235^ were unique to A/Egypt/N05056/09, which inferred these seven amino acids were the candidates for antigenic epitope according to the previous study [14].

**Table 3:** Amino acid residues in HA1 of A/Egypt/N05056/09 different from the other H5N1 viruses.

H5N1 virus	Genetic clade	Amino acid residue position a						
		22	120	151	152	154	210	235
A/duck/Hong Kong/p46/97	0	K	S	I	K	N	V	P
A/Vietnam/1194/04	1	K	S	I	K	N	V	P
A/Indonesia/5/05	2.1.3	K	S	I	K	N	V	P
A/Xinjiang/1/06	2.2	K	S	I	K	N	V	P
A/Egypt/N05056/09	2.2.1	R	N	T	Q	D	I	S
A/Anhui/1/05	2.3.4	K	S	I	K	N	V	P
A/common magpie/Hong Kong/2256/06	2.3.4	K	S	I	K	N	V	P
A/Japanese white-eye/Hong Kong/1038/06	2.3.4	K	S	I	K	N	V	P
A/goose/Guiyang/337/06	4	K	S	I	K	N	V	P

a Amino acid numbering is based on H5 HA protein.

To test the roles of these seven residues on antigen-antibody recognition of the H5 HA protein, we generated seven HA1 mutants based on wild type HA from A/Anhui/1/05 by site-directed mutagenesis. HA1 mutants with single amino acid mutation illustrated in Table 4 were expressed in 293T cells, and then immunofluorescence assays (IFA) were used to detect the interactions between the hH5M9 and the mutant HA1 proteins, while a rabbit polyclonal antibody against A/Anhui/1/05 was used as the positive control. As shown in Table 4, mutation of HA^P235G^ led to the abolishment of the binding ability of HA molecule to hH5M9 whereas other substitutions had no influence on antigen-antibody interaction. These results indicated that Pro^235^ in HA was critical for the binding to hH5M9.

**Table 4:** Epitope mapping of hH5M9 to site-directed mutant HAs from A/Anhui/1/05 by IFA.

Mutant site a	hH5M9	PC b
HA of wild type	+++ c	+++
K22G	+++	+++
S120G	+++	+++
I151G	+++	+++
K152G	+++	+++
N154G	+++	+++
V210G	+++	+++
P235G	-	+++
T231G	+++	+++
I232G	+++	+++
L233G	+++	+++
K234G	+	+++
N236G	-	+++
D237G	-	+++
A238G	+	+++
I239G	-	+++
N240G	-	+++
F241G	-	+++
E242G	+++	+++
S243G	+++	+++

a The number represented amino acid position in H5 HA.

b A rabbit polyclonal antibody against A/Anhui/1/05 was used as positive control (PC).

c IFA were performed on 293T cells transfected with mutant HA constructions. (+) to (+++) indicated the relative intensity of fluorescence.

To further identify the epitope localization, additional twelve HA site-mutants around Pro^235^ were generated and screened by hH5M9 (Table 4). Amino acid residues from 231 to 243 (except for 235) on HA were shifted to glycine individually by site-directed mutagenesis as described elsewhere [14]. As showed in Table 4, the mutants of HA^N236G^, HA^D237G^, HA^I239G^, HA^N240G^ and HA^F241G^ displayed complete loss of hH5M9 binding ability whereas mutations at HA^234^ and HA^238^ resulted in substantially reduction of binding ability to hH5M9. All newly constructed mutants had no impact on the binding to the rabbit polyclonal antibody against A/Anhui/1/05.

Thus, we concluded that the linear epitope targeted by CDR-grafted hH5M9 antibody was located from the amino acid residues 234 to 241 on the hemagglutinin of H5 subtype with the sequence of “Lys-Pro-Asn-Asp-Ala-Ile-Asn-Phe” (KPNDAINF). To further verify the epitope mapping, we have synthesized the KPNDAINF peptide coupled to KLH and determined the binding ability of hH5M9 to this peptide. The result indicated that hH5M9 interacted with peptide coupled to KLH whereas no response was detectable to KLH only (Figure 3B), which further demonstrated the existence of KPNDAINF epitope on H5 HA targeted by hH5M9 antibody. This epitope was supposed to be located on the top region of HA1 protein according to the published crystal structure of H5 HA (VN1194) (PDB No. 2IBX) (Figure 3C), which was never reported before. The illustration of the three dimensional structure (shown in Figure 3D) indicated that residues Asp^237^, Ala^238^, Ile^239^, Asn^240^, Phe^241^ comprised a sheet structure and residues Lys^234^, Pro^235^, Asn^236^ comprised a loop structure, with Lys^234^, Pro^235^, Asn^236^, Ala^238^ and Asn^240^ positioning on the outer face.

To determine the evolutionary conservatism of the identified epitope, we further aligned full-length sequences of HA from H5N1 virus strains available in the Influenza Research Database (http://www.fludb.org/brc/home.do?decorator=influenza) representing major epidemic avian H5N1 genetic clades up to Sep 2012. Among 2376 H5N1 virus strains, 1593 cases contained the epitope sequence identified in this study, indicating that hH5M9 was able to target near 67.0% of H5 strains reported. Moreover, among 243 human H5N1 virus strains, 179 cases contained this epitope with 73.7% coverage (Table 5). These data indicated that the epitope recognized by hH5M9 was conserved in epidemic avian H5N1 viruses, which made hH5M9 more extensive for its future application.

**Table 5:** The distribution of linear hH5M9 epitope among H5N1 virus strains from Influenza Research Database by Sep 2012.

Subtype	Epidemic Region	All species			Human		
		Strains with eptiope	Total	Detection rate	Strains with eptiope	Total	Detection rate
H5N1	All	1593	2376	67.0%	179	243	73.7%
H5N1	Asia	1253	1592	78.7%	173	183	94.5%
H5N1	Africa	108	513	21.1%	6	60	10.0%
H5N1	China	274	328	83.5%	34	39	87.2%
H5N1	Egypt	19	423	4.5%	6	60	10.0%
H5N1	Vietnam	236	293	80.5%	49	51	96.1%
H5N1	Indonesia	271	298	90.9%	10	10	100.0%
H1	All	0	11292	0.0%	0	9327	0.0%
H3	All	0	5969	0.0%	0	4564	0.0%
H6	All	0	945	0.0%	0	0	0.0%
H7	All	0	839	0.0%	0	7	0.0%
H9	All	0	894	0.0%	0	3	0.0%

## Discussion

With sporadic H5N1 avian flu outbreaks in poultry, wild birds and human, the emergence of a human-adapted H5 virus, either by reassortment or mutation, is a threat to public health worldwide. In the present study, we had successfully generated a CDR-grafted human antibody originated from high-affinity mouse hybridoma targeting HA with a spectrum of neutralizing activities against multiple strains of HPAI H5N1 virus in *vitro*.

To minimize the immunogenicity of murine antibodies, chimeric and CDR-grafting antibodies were generated. There are two approaches for CDR grafting [33], [34]. One strategy is to fix human FRs from heavy or light chain of representative antibodies. Another one is to choose frameworks of variable regions from human antibody that are the most homologous to those of the murine one for minimizing the distortion of CDRs. Here we have used the latter approach for humanization of mouse mH5M9 antibody against H5N1 virus. CDR-grafted hH5M9 was firstly constructed by directly grafting CDRs from VH and VL of mH5M9 to sequence-homologous VH and VL domain of human antibody FabOX108. Results from antigen-binding assay indicated that this led to the elimination of the binding activity (data not shown). This was not surprising due to the fact that the transfer of CDR residues alone into a xenogeneous antibody might alter the conformational structure of the antibody, leading to the decrease of antigen-binding affinity. Through three dimensional computational modeling, we chose eight amino acid residues in the FR regions of the VH chain of mH5M9 antibody for the substitution of CDR-grafted framework residues. Back mutation of four residues on heavy chain (Met^37^, Arg^66^, Val^71^, Val^109^) completely restored the binding avidity comparable to its mouse or chimeric counterpart. We thus deduced that the preservation of key mouse FR residues was critical for the successful design of CDR-grafted antibodies probably through constraining the CDR conformations [26].

Although antibody epitopes had been reported to be located in five of the eleven viral proteins of influenza virus, most of the epitopes were located in the viral HA [35]. For instance, antibodies 7H10 [13], AVFluIgG01, AVFluIgG03 [14], FLA5.10, FLD21.140 [36] or 65C6 [37] recognized HA1, while CR6261 bound to a highly conserved epitope on HA2 [38]. HA1 contained the receptor-binding site to host cells. The interference of interaction between HA1 and the receptor on host cells by functional antibodies largely protected the host from the infection of flu virus [20]. To date, five antigenic sites (A–E) on the H5 HA molecule had been mapped by using virus escape mutants (viral variants that could escape recognition by the monoclonal antibodies) [39], [40]. They were located exclusively in the areas corresponding to antigenic sites A and B of H3 HA and the antigenic site Sa of H1 HA [41], [42].

In the present study, hH5M9 was demonstrated to recognize a linear epitope of HA1 (amino acid residues 234–241, KPNDAINF), which was located on the top region of HA1 protein. Notably, this epitope was not listed in the reported five antigenic sites A–E [39], [40]. According to the three dimensional structure of H5 HA (VN1194) (Figure 3C), we found that this linear epitope (colored in green) comprised a remarkably tight cluster, far from the receptor-binding domain (RBD) of H5 HA1, as colored in yellow. We also aligned the sequences of 2376 H5N1 virus strains representing major epidemic avian H5N1 genetic clades, including 243 human clades. The results showed that the epitope was highly conserved in epidemic avian H5N1 viruses in clades 1–9 (Table 2), which was consistent with the broad cross-protection *in vitro* of hH5M9. The residues Lys^234^, Pro^235^, Asn^236^, Asp^237^, Ala^238^, Ile^239^, Asn^240^, Phe^241^ were highly conserved among the 2376 H5N1 virus strains. Interestingly, HA^Pro235Ser^ mutation existed in most Egypt clades belonging to clade 2.2.1. When we analyzed the conservation of this epitope in H5N1 strains reported annually, up to 94% of H5N1 strains had “KPNDAINF” before 2005. Inhibition on the viruses was of particular importance due to their wide geographical spread and pathogenicity in human. Based on our study, the antibody hH5M9 might counteract most of them. Meanwhile, similar epitope sequence recognized by hH5M9 in our study was found not to exist in any other HA subtypes. In other words, “KPNDAINF” was an H5N1-specific epitope. The H5 epitope for hH5M9 was also comprised in the H5N1 strain-specific hemagglutinin CD4^+^ T cell epitope [43]–[46]. More strikingly, it was not exactly the same epitope as parental mouse mH5M9 that was reported recently [47]. Considering that hH5M9 exhibited slightly decreased affinity to HA whereas the similar HI activity compared to the parental or chimeric H5M9, the difference between epitopes of parental mH5M9 and the CDR-grafted hH5M9 antibodies was supportive. Two possibilities might dedicate to the inconsistency of the epitopes recognized by parental or CDR-grafted antibody. One was that CDR grafting altered the amino acid compositions and the lengths of CDRs, which might change the conformation of variable regions and led to the subsequent shift of epitope recognition it used to [48]–[50]. Another was that the parental antibody might recognize two independent epitopes which had been reported in several studies [51]–[54]. We supposed that with its high affinity, conservation and specificity, this newly-defined linear epitope might take priority in peptide vaccine designing over conformational epitope against flu infection.

In summary, we had generated a CDR-grafted antibody that exhibited high neutralizing properties *in vitro* against H5N1 viruses. With the expected lower immunogenicity and the conservation of the epitope recognized by CDR-grafted antibody, we intended to expect that it might exhibit high neutralization breadth and potency *in vivo* and would be employed as adjunctive treatment against human H5N1 virus infection.

## Materials and Methods

### Materials

Mouse monoclonal antibody mH5M9 was elicited by immunization of mice with H5 HA concentrated from H5N1 virus (A/goose/Guangdong/1/96) and characterized as described previously [30]. mH5M9 antibody was purified from mouse ascites by caprylic acid-ammonium sulfate precipitation. The genes of heavy chain variable domain (VH) and light chain variable domain (VL) of mH5M9 were cloned from hybridoma by using reverse transcription-polymerase chain reaction (RT-PCR) routinely and subcloned in pMD19-T plasmid. The plasmids were gifted by Professor Xiaoyan Che from Central Laboratory of Zhujiang Hospital, the Southern Medical University (Guangzhou, China).

The HA proteins used in this study included: A/goose/Guangdong/1/96 (H5N1, clade 0), A/Vietnam/1194/04 (H5N1, clade 1), A/Indonesia/5/05 (H5N1, clade 2.1.3), A/Xinjiang/1/06 (H5N1, clade 2.2), A/Egypt/N05056/09 (H5N1, clade 2.2.1), A/Anhui/1/05 (H5N1, clade 2.3.4), A/common magpie/Hong Kong/2256/06 (H5N1, clade 2.3.4), A/Japanese white-eye/Hong Kong/1038/06 (H5N1, clade 2.3.4), A/goose/Guiyang/337/06 (H5N1, clade 4) and A/New Caledonia/20/99 (H1N1) (Sino Biological Inc., Beijing, China). In addition, inactivated influenza viruses were also used, including A/duck/Anhui/1/06 (H5N1, clade 2.3), A/chicken/Shanxi/2/06 (H5N1, clade 7), A/duck/Guangdong/1/96 (H7N3) and A/chicken/Shandong/6/96 (H9N2) (Weike Biotechnology Development Co., Harbin, China).

### Designing strategy of chineric and humanized H5M9 antibodies

To generate chimeric H5M9 antibody (cH5M9), the VH and VL gene fragments of mH5M9 monoclonal antibody in pMD19-T plasmids were subcloned into IgG expression cassette vectors IFH with the constant region of human antibody heavy chain (IFH-VH) and IFL with the constant region of human antibody κ light chain (IFL-VL), respectively. More detailed information of expression vectors was indicated in Figure S1.

To further humanize the mouse-human chimeric antibody, CDR-grafting method was used. We intended to choose human variable regions that were the most homologous to mouse variable regions as the templates for CDR grafting of mH5M9. Sequences of VH and VL domain from mH5M9 were subjected to a BLASTP search against the non-redundant Genbank database, respectively. VH and VL genes from FabOX108 were selected to construct humanized VH and VL regions by substituting CDR regions with mH5M9-derived CDR residues. The antibody structure was explicitly solvated with TIP3P water molecules using VMD [55], and then were subjected to molecular dynamics simulations. Simulations were performed with CHARMM force field using NAMD program [56]. The system was first minimized for 5000 steps with conjugate gradient method, and then was heated linearly to 300 K over 60 ps. The refined models were further assessed by VMD program. Humanized VH and VL fragment genes were fully synthesized (Generay Biotechnology, Shanghai, China) and subcloned into expression vectors.

### Expression and purification of chimeric or humanized antibodies

The recombinant heavy chain and light chain expression vectors, IFH-VH and IFL-VL, for chimeric or humanized antibodies were co-transfected into 293F cells using FreeStyle™ MAX Reagent (Invitrogen, USA) according to the manufacturer's instructions. The culture supernatant was subjected to ELISA assay to determine the expression and the antigen-binding capacity of the engineering antibodies. For purification of antibodies, the culture supernatant was subjected to Protein-A affinity chromatography on a HiTrap™ MabSelect SuRe column (GE Heathcare, Sweden). The integrity and purity of chimeric and humanized H5M9 antibodies were assessed by Coomassie blue staining after SDS-PAGE electrophoresis. Protein concentration was determined by RC DC Protein Assay (Bio-Rad, USA).

### ELISA

Indirect ELISA was performed to determine the specificity of antibodies. Briefly, the samples were added to 96-well plates pre-coated with 1 µg H5 HA overnight and incubated at 37°C for 1 h. After washing, 100 µl of horseradish peroxidase- conjugated goat anti-human IgG (KPL, USA) or goat anti-mouse IgG (Jackson ImmunoResearch, USA) were added to each well and incubated at 37°C for 1 h. Finally, 100 µl of 0.2 M citrate buffer (pH 5.0) containing 0.04% o-phenylenediamine (Amresco, USA) and 0.03% H_2_O_2_ were added to each well and incubated for 10 min. The reaction was stopped by the addition of 50 µl 2 M H_2_SO_4_, and the optical density was measured at 490 nm by an ELISA plate reader (Bio-Rad, USA).

In the experiment for epitope verification, 1 µg of H5 HA from A/Vietnam/1194/04 strain, synthesized KPNDAINF coupled to KLH or KLH only were coated overnight. Diluted hH5M9 antibody were added to the wells and incubated at 37°C for 1 h. Further detection was performed as described above. The peptide containing a C-terminal cysteine for conjugation to KLH was synthesized in Sangon Biotechnology (Shanghai, China).

### Hemagglutination inhibition (HI) assay

To determine the biological activity of recombinant hH5M9, the classical HI assays were performed as described elsewhere [57]. Briefly, the purified antibodies were 2-fold diluted in 96-well plates and mixed with 4 HA units of H5 viruses, including A/goose/Guangdong/1/96 (H5N1), A/Vietnam/1194/04 (H5N1), A/duck/Anhui/1/06 (H5N1), A/Anhui/1/05 (H5N1), A/chicken/Shanxi/2/06 (H5N1), A/New Caledonia/20/99 (H1N1), A/duck/Guangdong/1/96 (H7N3) and A/chicken/Shandong/6/96 (H9N2). HI capacities against H1, H7, and H9 viruses were simultaneously determined. Plates were incubated for 30 min at room temperature, and 1% of chicken red blood cells (RBCs) were added to each well. The initial concentration of each antibody was 1 mg/ml. The highest dilution of antibodies in which agglutination was not observed was considered to be the hemagglutination inhibition endpoint.

### Affinity measurement by Surface Plasmon Resonance

Surface plasmon resonance (SPR) analysis of hH5M9 was performed on ProteOn surface plasmon resonance biosensor (BioRad Labs, Shanghai, China). The H5 HA (A/Vietnam/1194/04) was immobilized on a GLC sensor chip using an amine coupling kit with 946 resonance units (RU) in the testing flow cells. 200 µl of freshly prepared antibodies at various concentrations were injected at a constant flow rate of 100 µl/min with 120 s duration at 25°C. Responses from the HA surface were normalized by that from the mock surface without protein binding or a separate injection with buffer only. An anti-EGFR antibody C225 was used as a negative control. Binding kinetics for the antibodies and the data analysis were performed using ProteON manager software (Bio-Rad, USA).

### Western blotting

H5 HA (A/Anhui/1/05) was applied to 12% SDS-PAGE and transferred to a PVDF membrane with a Trans-Blot SD semi-dry transfer cell apparatus (Bio-Rad, USA). hH5M9 (1 mg/ml) was used as primary antibody. A rabbit polyclonal antibody against A/Anhui/1/05 was used as the positive control. IRDye 800CW-conjugated goat anti-human or goat anti-rabbit IgG (LI-COR, USA) was used as the secondary antibody for visualization. The fluorescent bands were detected by Odyssey Imaging System (LI-COR, USA).

### Construction of HA mutants by site-directed mutagenesis

Mutagenic primers were designed by software Primer Premier 5.0 (Premier Biosoft International, USA) after sequences alignment of HA region from H5N1 isolates including A/goose/Guangdong/1/96 (Genbank accession AAD51927), A/duck/Hong Kong/p46/97 (AAF02306), A/Vietnam/1194/04 (ACR48874), A/Indonesia/5/05 (AFM78567), A/Xinjiang/1/06 (ACJ68614), A/Egypt/N05056/09 (ACT15357), A/Anhui/1/05 (ADG59080), A/common magpie/Hong Kong/2256/06 (ABJ96777), A/Japanese white-eye/Hong Kong/1038/06 (ABJ96775) and A/goose/Guiyang/337/06 (ABJ96698). Nineteen amino acid residues in HA gene of A/Anhui/1/05 (Lys^22^, Ser^120^, Ile^151^, Lys^152^, Asn^154^, Val^210^, Thr^231^, Ile^232^, Leu^233^, Lys^234^, Pro^235^, Asn^236^, Asp^237^, Ala^238^, Ile^239^, Asn^240^, Phe^241^, Glu^242^ and Ser^243^) were site-mutated to glycine (G) by PCR. The site-directed mutant fragments were digested by *Bam*HI and *Xho*I (Takara, Japan) and subcloned into pcDNA3.0 vector. All constructs were confirmed by DNA sequencing and subjected to transfection for transient expression of mutant HA molecules in 293T cells. Immunofluorescence assays were subsequently performed to determine the binding ability of mutant HA molecules to hH5M9.

### Immunofluorescence Assay (IFA)

IFA were performed on 293T cells transfected with mutant HA constructions. Cells were grown in 24-well plates, fixed in 3.7% paraformaldehyde, permeabilized with 0.1% Triton X-100 (Sigma-Aldrich, USA), and then stained in the wells. Purified hH5M9 (1 mg/ml) was used as primary antibody. A rabbit polyclonal antibody against A/Anhui/1/05 was used as the positive control. Binding antibodies were detected by using Cy2-conjugated goat anti-human or goat anti-rabbit IgG (Jackson ImmunoResearch, USA) and observed under immunofluorescence microscope (Olympus, Japan).

## Supporting Information

Figure S1 **Structure of expression cassette vectors IFH with the constant region of human antibody heavy chain (A) and IFL with the constant region of human antibody κ light chain (B).** Variable region of heavy chain was cloned into *Afl*II and *Nhe*I sites of IFH vector. Variable region of light chain was cloned into *Eco*RV and *Hin*dIII sites of IFL vector.
(TIF)

Figure S2 **Molecular modeling of mH5M9 variable regions.** The CDRs were shown in red and the FRs were shown in blue. Eight framework residues, H24, H37, H67, H71, H93, H109, L58 and L87, which were different from FabOX108 while critical for constraining the CDR conformations, were colored in green.
(TIF)
