# Supplementary figures and images for: A High-Affinity CDR-Grafted Antibody against Influenza A H5N1 Viruses Recognizes a Conserved Epitope of H5 Hemagglutinin
Source: PLoS One. 2014 Feb 18;9(2):e88777. doi: 10.1371/journal.pone.0088777 (PMC3928294; doi:10.1371/journal.pone.0088777)

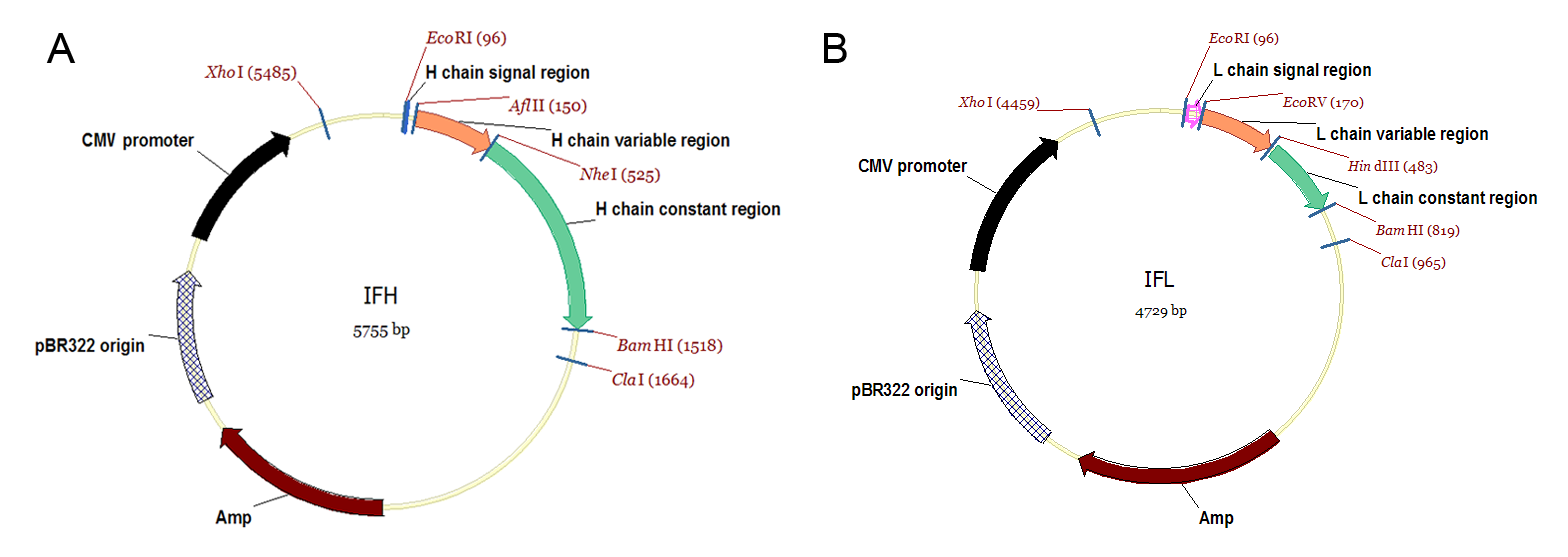

Supplement: Figure S1 — Structure of expression cassette vectors IFH with the constant region of human antibody heavy chain (A) and IFL with the constant region of human antibody κ light chain (B). Variable region of heavy chain was cloned into AflII and NheI sites of IFH vector. Variable region of light chain was cloned into EcoRV and HindIII sites of IFL vector. (TIF) [file pone.0088777.s001.tif]

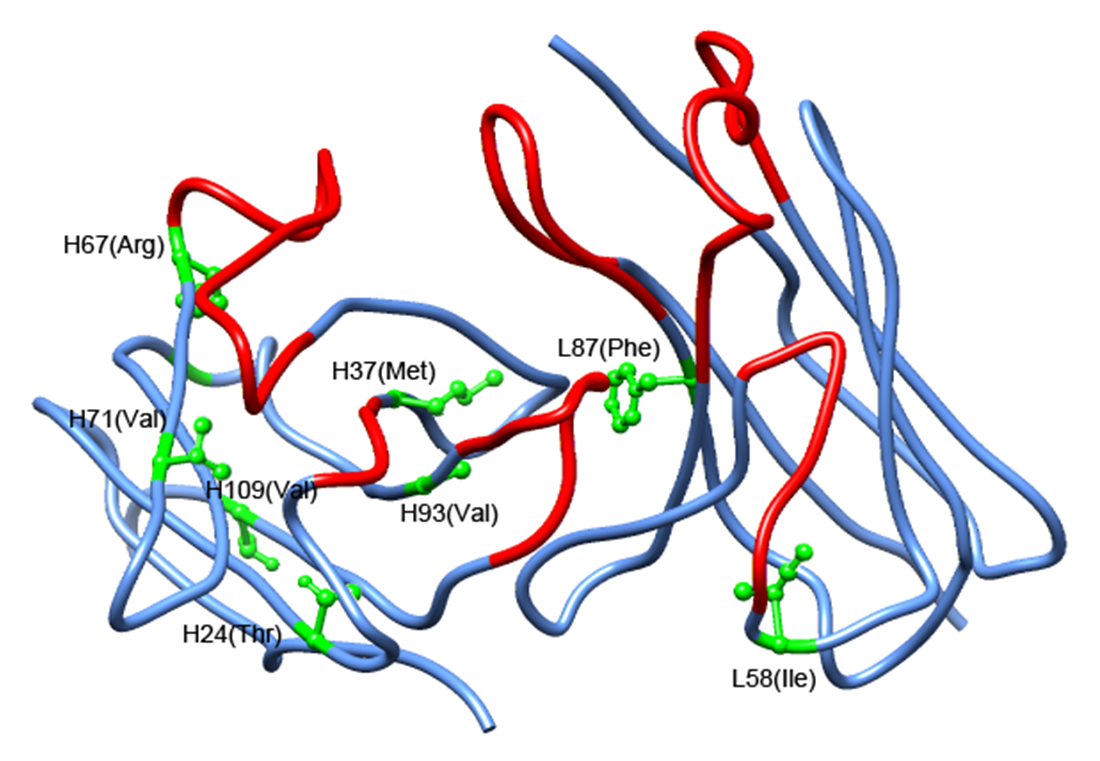

Supplement: Figure S2 — Molecular modeling of mH5M9 variable regions. The CDRs were shown in red and the FRs were shown in blue. Eight framework residues, H24, H37, H67, H71, H93, H109, L58 and L87, which were different from FabOX108 while critical for constraining the CDR conformations, were colored in green. (TIF) [file pone.0088777.s002.tif]
